# Maternal dietary diversity and nutritional adequacy in relation with anthropometric measurements of newborns at birth: a cohort study in Tehran city

**DOI:** 10.1186/s12887-021-03102-3

**Published:** 2022-03-12

**Authors:** Tahereh Karimi, Hassan Eini-Zinab, Arezoo Rezazadeh, Zeinab Moslemi

**Affiliations:** Department of Community Nutrition, National Nutrition and Food Technology Research Institute, Faculty of Nutrition Sciences and Food Technology, Shahid Behehshti University of Medical Sciences, Tehran, Iran

**Keywords:** Diet diversity and adequacy, Pregnancy, Neonatal, Anthropometric characteristics, Cohort study

## Abstract

**Background:**

Maternal dietary intake during pregnancy plays an important role in fetal development and birth outcomes. The aim of the present study was to determine maternal dietary diversity and Nutritional adequacy in relation with anthropometric measurements of newborn at birth as a cohort study in Tehran city.

**Methods:**

This prospective cohort study, was conducted by participation of 585 pregnant women referred to public health centers and hospitals covered by Shahid Beheshti, Tehran and Iran Universities in Tehran City. Using face-to-face interviews, general characteristics were obtained by questionnaire. Pre-pregnancy dietary intake was measured by a 168-item semi-quantitative food frequency questionnaire at the first visit, and dietary intake during pregnancy was measured by 2 non-consecutive 24-h food recall (one holiday and one regular day) at 31–34 weeks. Maternal height and weight were measured using standard tools and protocol at the first visit, and maternal weight at the end of pregnancy and data related to neonatal anthropometric indices were collected from mothers and neonates health records in the *Sib* electrical system. By applying SPSS software (version 23) the association was analyzed by linear regression with adjusting for confounding factors. *P*-value< 0.05 was considered as significant.

**Results:**

Mean ± standard deviation of body mass index (BMI) of pre-pregnancy, pregnancy weight gain, BMI for age z-score (BAZ) at birth of infants were 24.52 ± 4.12, 12.16 ± 6.85 kg and − 0.61 ± 1.48, respectively. Mean ± SD of the Dietary Diversity Score (DDS) and Mean Adequacy Ratio (MAR) before and during pregnancy were 5.31 ± 1.11, vs.5.23 ± 1.42 and 289.85 ± 113.12 vs. 371.07 ± 197.28, respectively. After adjusting for confounding factors DDS in the third trimester of pregnancy was inversely correlated with WAZ (B = -0.16, 95% CI = - 0.23_0.30) and BAZ (B = − 0.24, 95% CI = - 0.06_0. 42) at birth, MAR of pre-pregnancy (B = − 0.001, 95% CI = - 0.002_0.00) and in the third trimester of pregnancy (B = − 0.18, 95% CI = - 0.35_0.004) were negatively associated with WAZ at birth.

**Conclusion:**

The findings showed that maternal nutritional status (dietary diversity and nutritional adequacy) before and during pregnancy were correlated with neonatal anthropometric indices at birth.

## Background

Maternal dietary intake during pregnancy plays a vital role on fetus growth and birth outcomes [1–6]. Mother’s nutritional status is an important marker of infant’s survival and health outcomes such as chronic disease risk during their childhood and adult life [7–9]. Although animal studies have consistently demonstrated a strong positive relationship between adequate maternal nutrition and birth outcomes, this relationship is much less consistent in humans. This inconsistency maybe due to a confounding effects of number of environmental factors such as cultural background and socioeconomic differences [10]. Pregnant women are vulnerable groups for malnutrition risk particularly those living developing countries that a significant number of expectant mothers do not receive optimal level of essential nutrients during their pregnancy due to socio-economic constraints, frequent reproductive cycles and poor dietary adequacy [8, 11–14]. Also, diet diversity which is shown as a proxy of food security [15], may be a predictor of mother’s dietary sufficiency [10, 16, 17]. Dietary diversity score (DDS), is a simple, rapid and useful tool used as an indicator for assessing the adequacy of nutrient and energy intake, diet quality and reflects the consumption of various foods between and within each foods group [18–21].

Evidences showed poor maternal diet was related with malnutrition and low total pregnancy weight gain that is strongly related with nonoptimal anthropometric measures of newborn such as low birth weight [12]. However, previous studies did not reveal similar results. As an instance, findings of a study showed that higher maternal carbohydrate intake in the second trimester of pregnancy had a significant positive relationship with maternal weight gain and birth weight [22].while, in another study, maternal carbohydrate intake was not related with birth weight but it was inversely related to infant height [1]. In the other hand, there is few results exploring the relation between maternal diet diversity and anthropometric measures of newborns and majority of the studies that investigated the association between maternal nutrition with newborn size have approached only neonatal weight as a parameter of fetal growth [21, 23] and other measures such as head circumference and indices such as body mass index (BMI) was less addressed.

Studies conducted in Iran indicate that the mean intake of nutrients by pregnant women was less than optimal [24]. There was no study to find out the Iranian mothers’ dietary diversity in pregnancy status in relation with birth anthropometric measures as a prospective follow-up study. So, the aim of the present study, was to investigate the relationship between maternal dietary diversity score and nutritional adequacy with anthropometric indices of newborns of Iranian women living in Tehran city: a prospective cohort study.

## Method

This cohort study was performed on 585 pregnant women referring to public health centers and hospitals under-coverage of Shahid Beheshti, Tehran and Iran universities between Decembers 2015_ may 2018. Samples were selected by simple random sampling method. All pregnant women in their first trimester of pregnancy were recruited from public health facilities for first interview.

Criteria for entering the study included being at < 22 weeks of gestation, tending to cooperate with the project and for newborns including live and healthy cases born from studied pregnant mothers. Exclusion criteria was included the compliance of a pregnant mother with a particular diet, unwillingness to continue the collaboration with the project, abortion and miscarriage, over and under reporting of energy intake outside the range of mean ± 3 standard deviation and birth of infants with severe conditions such as physical or mental retardation.

The sample size was calculated using an odds ratio observed between maternal consumption of vegetables and birth weight [19]. The sample size formula for the method described by Kelsey et al. is:

$${\mathbf{n}}_{\mathbf{1}}=\frac{{\left({\mathrm{z}}_{\mathrm{w}2}+{\mathrm{z}}_{1-\beta}\right)}^2\mathrm{pq}\left(\mathrm{r}+1\right)}{\mathrm{r}{\left({\mathrm{p}}_1-{\mathrm{p}}_2\right)}^2}$$

and

$${\mathrm{n}}_2=\mathrm{r}\kern0.5em {\mathrm{n}}_1$$

where

**n**_**1**_ = _number of cases_

**n**_**2**_ = _number of controls_

**z**_**w2**_ = standard normal deviate for two-tailed test based on alpha level (relates to the confidence interval level)

**z**_**β**_ = standard normal deviate for one-tailed test based on beta level (relates to the power level)

r = ratio of controls to cases

p_1_ = proportion of cases with exposure and q_1_ = 1-p_1_

p_2_ = proportion of controls with exposure and q_2_ = 1-p_2_

$$\mathrm{p}=\frac{\mathrm{P}1+{\mathrm{r}\mathrm{p}}_2}{\mathrm{r}+1}$$

and

$$\mathbf{q}=\mathbf{1}-\mathbf{p}$$

**Fig. 1:**
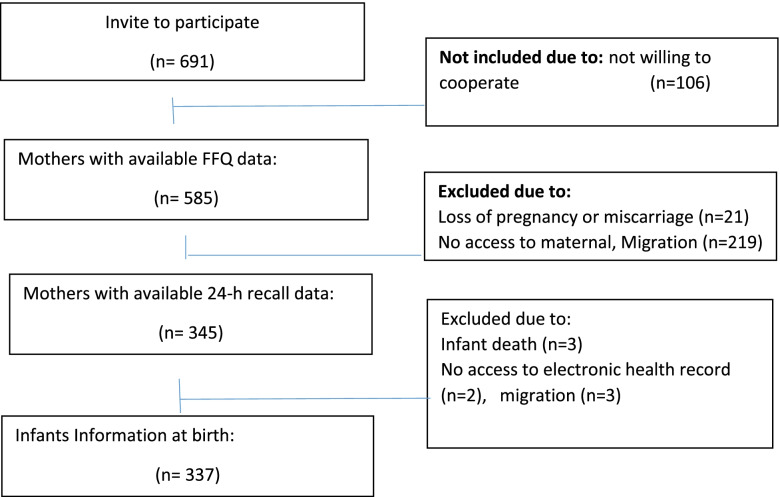
Flow chart of the recruitment process

Where, Alpha = 0.05, power (1 – beta) = 80% and OR = 3.75. The calculated sample size was 184 for public and Private (clinics and hospitals) health centers, separately [*n* = 368]. Considering the higher possibility of attribution in cohort studies, we recruited about two times the calculated samples) *n* = 691) in order to take into account possible attritions which we assumed it to be 50%. The recruitment process was described in detail in Fig. 1.

In brief, of 691 invited women, 585 women agreed to participate in the study at first stage interview; for the second stage, only 342 of them were available/willing to cooperate. Other reasons for non-participation was loss of pregnancy (e.g. miscarriage) and migration. At the third stage, maternal information of 345 women and 337 newborns extracted from health records. Additional reasons for data attrition at the last stage was lack of information on birth weight and stillbirth. Due to the fact that, final number of live births were 337, only maternal information of these babies was used for analyzing the association between maternal intake and newborns anthropometric outcomes.

To be ensure that baseline information of those mothers remained for final analysis were not different from the total baseline participants, it was checked if there were any significant differences, in terms of both dependent (pre-pregnancy BMI) and the main independent variables (such as age, socioeconomic status, energy intake, pre-pregnancy DDS and MAR, between 337 remained women and those who were eliminated from the study and there was not observed any significant differences (*p* > 0.05) [data was not represented in the tables].

The collected data in the first stage included pregnancy history, pre pregnancy dietary intake (by Food Frequency Questionnaire (FFQ)), socioeconomic status, medical history, and other mother-related factors related to birth weight. These data were collected through face-to-face interviews with pregnant mothers in the first trimester of pregnancy. The second interview included assessing mother’s current dietary intake by two 24-h dietary recall (24-h DR) at third trimester. The third stage of data collection included gathering mother and infant information from health facilities routine registration system at the end of third trimester of mother and new born birth. The procedure of variables assessment was described in detail as follows:

### Dietary assessment

Dietary intake data were collected by trained nutritionist. Maternal usual dietary intake of pre-pregnancy was assessed using food frequency questionnaire (FFQ) using a validated semi-quantitative FFQ consisting 168 food items (with standard serving sizes) through face to face interviews in the first interview (< 22 weeks pregnancy). Although FFQ was obtained in < 22 weeks of pregnancy, women were asked to remember frequency of their usual intake during past years assuming when they were not pregnant. The FFQ was designed according to Willet’s method [25]. The reliability and validity of the questionnaire was evaluated earlier using a sample of adult women in Tehran [26]. For each food item, participants reported the consumption frequency according to suggested portion size during the past year. Actual Dietary intake of mothers at third trimester of pregnancy measured using two non-consecutive 24_h recall including food consumption for a typical week day and one weekend day collected at 31-34th weeks of their gestation. Afterward, reported dietary intake by both dietary assessment methods were converted to daily consumption in grams by using household measures [27]. Mean intake of two 24-h DR was considered for second stage analysis. Total daily energy intake was calculated using the information from USDA food composition table (USDA, Release 11, 1994) adapted for Iranian food products [28].

### Diet diversity and nutritional adequacy assessment

Diet diversity of mother’s diet in pre-pregnancy and pregnancy status. Kant et al. have developed a method for scaling dietary diversity using a variety of food groups [29]. In this method, the categorization of food groups according to the definition of the food pyramid, were used 5 groups of bread and cereals, vegetables, fruits and dairy products meat and its alternatives. The main groups were divided into 23 subgroups (7 for bread and cereals, 7 for vegetables, 2 for fruits, 3 for dairy products and 4 for meat and alternatives). These subgroups were defined to include diversity of food items in each food group of the pyramid [30]. The division of bread and cereals into seven subgroups shows the importance of diversity in the consumption of cereal-based foods. In order to be considered as a consumer of a food subgroup, at least half of the defined portion of that food must be consumed in accordance with the definitions of the quantitative indicators of the food pyramid in the 2 days of recall. Certainly, a higher score and close to 10 indicates a better compliance with the diversity principle based on the recommendations of the food pyramid. Of course, the probability that a person will use all 23 subgroups of the pyramid within 2 days is very low and studies have shown that perhaps only 10% of the population can use all 23 subgroups within 2 days [31].

Nutrient adequacy ratios (NARs) were calculated for the intake of energy and the 11 nutrients (vitamin A, riboflavin, thiamine, vitamin C, calcium, iron, zinc, Phosphorus, magnesium, protein and potassium) based on the recommended daily allowance (RDA) for that nutrient. In the present study, it was used the medical institution’s RDA for the different age groups of pregnant women [32]. The average of the food adequacy ratio was based on the formula in the Krebs-Smith study and the indicator was calculated by dividing the amount of nutrients and energy consumed by the amount of their recommended amount [33]. MAR was also calculated as the sum of NARs for all assessed nutrients divided by the number of assessed nutrients. To assess an overall estimate of the nutritional adequacy a mean adequacy ratio (MAR) was calculated from the 11 NARs according to the following formula:

$$MAR=\frac{\sum NAR}{\mathrm{Number}\kern0.5em \mathrm{of}\kern0.5em \mathrm{nutrients}}$$

### Assessment of other variables

Data on maternal demographic (age, marital status, pre-marital familial relationship with spouse, number of family members, religion, ethnicity, and nationality), socioeconomic status (education, employment status, household income) were collected by a valid questionnaire [34]. Other variables included physical activity assessed by International Physical Activity Questionnaire validated for Iranians (IPAQ) [35], general health status assessed by validated GHQ-28 questionnaire [36]. Pregnancy complications, past medical history and abortion, number of prenatal care, pregnancy number, and gestational age were also collected by question and referring to the electronic health records (*Samaneh Sib*).

### Maternal and neonatal anthropometric measures

Pre-pregnancy weight was obtained from the health registry system. Mother’s weight and height were measured at first interview, as well. Weight was measured to the nearest 100 g without shoes and with minimal clothes. Height was measured to the nearest 1 mm without shoes and standing straight with normal position of shoulders. Mother‘s Body Mass Index was calculated as weight (in kilograms) divided by height (in meters) squared. Mother’s weight at third trimester obtained from health records after making adjustments for differences in measurement tools. The health facility scales were calibrated using research team scales. In the final stage, information of born infants including anthropometric indices of infant, gender, date of birth and type of delivery were collected by referring the researcher to health centers using the infant’s electrical health record named *Samaneh Sib.*. If information was not available in the electronic database, the mothers were interviewed using telephone calls and the necessary information was collected. Infant WAZ, HAZ and BAZ were calculated using gender-specific reference database form the World Health Organization (WHO) AnthroPlus for personal computers software, version 1.0.4) [37].

### Statistical analysis

Data was analyzed using SPSS software (version 21). Both univariate and multivariate analysis were conducted. For univariate analysis, frequency and percentage or mean and standard deviation was used depending on the measurement type. The relationships between maternal dietary diversity and nutritional adequacy before and during pregnancy with anthropometric measurements of newborn at birth were analyzed at both pre-pregnancy and third trimester. Unadjusted and adjusted linear regression models were used to assess the relationships. Dependent variable for both unadjusted and adjusted linear models was birth weight.

From the total 691 women recruited at first stage interview, 585 women completed all questionnaires of the first stage (Fig. 1) of whom, 345 were available/willing to participate at second interview and completed the 24-h recall. At the third stage, information of 337 infants’ anthropometric measurements (considering all participating mothers at the first stage who delivered alive and healthy baby) at birth were obtained from electronic health records called *Samaneh Sib*. Infants’ information is routinely recorded in that electronic system by health care givers.

## Results

Socioeconomic and demographic characteristics of the samples are presented in Table 1. Mean ± SD of mothers age was 28.76 ± 6.13 years with the range between 16 and 46 years old. Most of mothers were 18–35 years old, housewives with low or medium educational level, living in household with size 4 or less, and about one-third of them had no prior familial relationship with their husband and more than two thirds of them lived in households with lower than the mean monthly income.

**Table 1:** Socioeconomic and demographic characteristics of the sample at first phase of the study

Characteristics	Frequency (percent)	
Mother’s Age (years)	< 18	17 (2.9%)
	18–35	464 (79.3%)
	> 35	104 (17.8%)
Mother Education	Low	176 (30.1%)
	Medium	249 (42.5%)
	University degree	160 (27.4%)
Employment Status of the Mother	Housewife	532 (90.9%)
	Employed	53 (9.1%)
Prior Familial Relationship	Relative	204 (34.9%)
	Non-Relative	381 (65.1%)
Number of household Members	≤ 4	560 (95.7%)
	≥5	25 (4.3%)
Household Income/month (Dollar)	Less 300 Dollar	295 (50.4%)
	> = 300 Dollar	290 (49.6%)

Maternal and neonatal anthropometric indices, energy intake, diversity score, and nutritional adequacy of the women studied are shown in Table 2. The results show that the average energy intake of mothers at the third trimester of pregnancy was lower than pre-pregnancy. The mean score of mother’s dietary diversity was also reduced during pregnancy, but the mothers’ average nutritional adequacy was increased in the third trimester. Pregnancy Outcomes included delivery time in weeks, type of delivery, gender, number of delivered newborns and birth weight (gr) are shown in Table 3. The results show that the more than half of the women underwent cesarean section and 38% of infants were low birth weight (< 2500 g).

**Table 2:** Maternal and neonatal anthropometric indices in the studied mothers and infants

Variable (unit)	Mean ± SD
Prenatal weight (kg)	63.40 ± 11.07
Mother height (cm)	160.80 ± 5.95
Pre-pregnancy BMI (kg / m 2)	24.52 ± 4.12
Maternal weight gain during pregnancy (kg)	12.16 ± 6.85
Pre-pregnancy energy intake (Kcal/day)	2781.30 ± 1054.37
Pregnancy (third trimester) energy intake (Kcal/day)	2755.97 ± 989.46
Pre-pregnancy Diversity Score	5.31 ± 1.11
Third trimester of pregnancy DDS	5.23 ± 1.42
Pre-pregnancy MAR	289.85 ± 113.12
Pregnancy MAR pregnant	371.07 ± 197.28
Birth weight (gr)	3179.87 ± 486.96
Birth height (cm)	49.76 ± 2.20
WAZ	−0.32 ± 1.08
HAZ	0.13 ± 1.92
BAZ	−0.61 ± 1.48
HCZ	−0.11 ± 1.56

*DDS* Dietary Diversity Scores, *MAR* Mean Adequacy Ratio, *WAZ* Weight for Age Z-score, *HAZ* Height for Age Z-score, *BAZ* BMI for Age Z-score, *HCZ* Head Circumference for age Z-score

**Table 3:** Pregnancy outcome characteristics of studied mothers and their newborns

Characteristics	Frequency (percent)	
gestational week	Pre-term (< 37 weeks)	39 (8.6%)
	term (37–42 weeks)	391 (85.7%)
	Post-term (> 42)	26 (5.7%)
Type of delivery	Natural	212 (43.9%)
	CS	271 (56.1%)
Gender	Female	232 (47.8%)
	male	253 (52.2%)
Type of pregnancy	singleton	469 (96.7%)
	Twin	16 (3.3%)
Birth weight (gr)	< 2500 g	38 (7.8%)
	2500–4000 g	430 (88.7%)
	> = 4000 g	17 (2.9%)

*CS* Caesarean section

Mean scores of food groups according to the total DDS tertiles of pre-pregnancy and third trimester of gestation of studied mothers are presented at Table 4. Findings showed that there was a significant difference between pre-and post-pregnancy dietary diversity for bread and cereals, meat, dairy and vegetables. But for the fruit group, women in the first tertile had a significant difference with women in the second and third tertiles, but no significant difference was found between the second and third tertiles.

**Table 4:** Mean (standard deviation) of food groups scores according to the total DDS tertiles of pre-pregnancy and third trimester of gestation of studied mothers

Pre-pregnancy (*n* = 337)					Third trimester of pregnancy (*n* = 337)			
	First tertile	Second tertile	Third tertile	*P* value^1^	First tertile	Second tertile	Third tertile	*P* value^1^
grain score^a,b,c^	0.92 (0.25)	1.07 (0.24)	1.15 (0.23)	< 0.001	0.66 (0.23)	0.79 (0.22)	0.98 (0.26)	< 0.001
vegetable score^a,b,c^	0.65 (0.30)	0.91 (0.29)	1.12 (0.29)	< 0.001	0.55 (0.38)	0.78 (0.33)	1.04 (0.32)	< 0.001
meet score^a,b,c^	0.21 (0.28)	0.45 (0.33)	0.76 (0.38)	< 0.001	0.51 (0.38)	0.88 (0.39)	1.11 (0.40)	< 0.001
dairy score^a,b,c^	0.71 (0.53)	1.01 (0.48)	1.40 (0.43)	< 0.001	0.72 (0.51)	1.18 (0.42)	1.64 (0.39)	< 0.001
fruit score^a,c^	1.76 (0.52)	1.98 (0.13)	2.00 (0.00)	< 0.001	1.13 (0.68)	1.84 (0.35)	1.96 (0.17)	< 0.001

^1^*P*-value was obtained by one-way ANOVA. The significance between tertiles was obtained by Post-hoc analysis (Tukey, LSD)

^a^Significant difference between first and third tertile

^b^Significant difference between second and third tertile

^c^Significant difference between first and second tertile

Association of before and during pregnancy DDS with infants’ anthropometric indices is shown in Table 5. The results showed that there was no significant relationship between pre-pregnancy maternal DDS and neonatal anthropometric indices with moderating effect of confounding variables. But there was a significant inverse relationship between maternal DDS during pregnancy and weight, WAZ and BAZ of neonate.

**Table 5:** Comparison of an average intake of energy, micronutrients and macronutrients for pre-pregnancy and third trimester of pregnancy in mothers in the study^*^

	Pre-pregnancy *n* = 337		During pregnancy *n* = 337		
	Mean ± SD	RDA	Mean ± SD	RDA	*P*-value
**Energy (Kcal/d)**	2729.6 ± 895.11		2744.71 ± 935.46	2855	0.82
**Carbohydrate (g/d)**	405.09 ± 143.12		382.08 ± 140.49	175	**0.03**
**Protein (g/d)**	99.03 ± 36.11		131.85 ± 84.47	71	**< 0.001**
**Fat (g/d)**	95.41 ± 39.70	–	99.36 ± 43.88	–	0.21
**Cholesterol (mg/d)**	294.43 ± 149.06	–	345.85 ± 226.25	–	**< 0.001**
**SFA (g/d)**	29.66 ± 12.55	–	29.48 ± 13.55	–	0.85
**PUFA (g/d)**	26. 40 ± 14.60	–	20.01 ± 13.08	–	**< 0.001**
**MUFA (g/d)**	44.79 ± 18.47	–	54.13 ± 26.80	–	**< 0.001**
**Iron (mg/d)**	18.85 ± 6.65	18	22.32 ± 15.64	27	**< 0.001**
**Calcium (mg/d)**	1204.62 ± 439.35	1000	1407.62 ± 591.59	1000	**< 0.001**
**Sodium (mg/d)**	8557.07 ± 3720.44	1500	4238.09 ± 1667.52	1500	**< 0.001**
**Zinc (mg/d)**	10.05 ± 4.44	8	17.94 ± 10.62	11	**< 0.001**
**Magnesium (mg/d)**	327.24 ± 123.29	320	382.36 ± 195.85	350	**< 0.001**
**Phosphorus (mg/d)**	1470.97 ± 635.87	700	1383.88 ± 667.49	700	0.06
**Potassium (mg/d)**	3939.83 ± 1432.59	2600	3559.80 ± 1546.32	2900	**0.001**
**Iodine (μg/d)**	2.56 ± 4.42	150	0.28 ± 4.41	220	**< 0.001**
**Vitamin A (μg/d)**	1123.35 ± 638.82	700	1119.09 ± 860.38	770	0.92
**Thiamine (mg/d)**	2.35 ± 1.15	1.1	2.51 ± 1.62	1.4	0.12
**Riboflavin (mg/d)**	20.36 ± 30.61	1.1	25.28 ± 35.22	1.4	**0.04**
**Vitamin B6 (mg/d)**	4.45 ± 5.42	1.3	1.69 ± 5.49	1.9	**< 0.001**
**Folate (μg/d)**	628.73 ± 226.41	400	613.15 ± 247.66	600	0.37
**Vitamin B12 (μg/d)**	13.64 ± 27.01	2.4	8.57 ± 2.67	2.6	**0.01**
**Vitamin C (mg/d)**	191.55 ± 106.83	75	162.43 ± 122.04	85	**0.001**
**Vitamin D (μg/d)**	9.60 ± 12.32	15	9.90 ± 8.55	15	0.82
**Vitamin E (mg/d)**	7.55 ± 4.36	15	7.99 ± 7.38	15	0.34

* *P*-value was obtained using paired *t*-test and *P* < 0.05 was considered as significant

*RDA* Recommended Dietary Allowance, *SFA* Saturated Fatty Acids, *PUFA* Polyunsaturated Fatty Acids, *MUFA* Monounsaturated Fatty Acids

According to the study findings, the mean carbohydrate, PUFA, sodium, vitamins B6, B12, and vitamin C intake of pre-pregnancy were significantly higher than the third trimester. While, the mean intake of protein, cholesterol, MUFA, iron, calcium, zinc and magnesium was increased significantly during pregnancy (Table 6).

**Table 6:** Relationship of maternal dietary diversity score and nutritional adequacy before and during pregnancy with anthropometric measurement of newborn at birth^*^

		DDS				MAR			
		Pre-pregnancy (*n* = 337)		Third trimester of pregnancy (*n* = 337)		Pre-pregnancy (*n* = 337)		Third trimester of pregnancy (*n* = 337)	
		B(95%CI)	*P*-value	B (95%CI)	*P*-value	B (95%CI)	*P*-value	B (95%CI)	*P*-value
Weight	Crude model^a^	18.16 (−22.32_58.64)	0.37	−14.36 (−51.48_22.76)	0.44	−29.34 (−83.73_25.04)	0.29	−53.5 (− 116.2_9.12)	0.09
	Adjusted Model^b^	−2.65 (−74.00_68.70)	0.94	−76.93 (− 142.18_11.68)	**0.02**	−0.54 (− 1.09_0.004)	**0.05**	−98.51 (− 181.94_-15.08)	**0.02**
Height	Crude model^a^	0.03 (− 0.15_0.21)	0.73	0.008 (− 0.18_0.16)	0.92	− 0.17 (− 0.41_0.07)	0.17	− 0.22 (− 0.51_0.07)	0.14
	Adjusted Model^b^	0.02 (− 0.26_0.31)	0.86	− 0.009 (− 0.3_0.28)	0.95	− 0.001 (− 0.004_0.001)	0.27	−0.38 (− 0.76_0.003)	0.05
CH	Crude model^a^	0.06 (−0.09_0.23)	0.40	0.04 (−0.11_0.2)	0.57	−0.28 (− 0.5_-0.07)	**0.009**	− 0.17 (0.44_0.09)	0.21
	Adjusted Model^b^	0.13 (−0.02_0.29)	0.25	0.06 (−0.24_0.36)	0.67	−0.25 (− 0.59_0.07)	0.12	− 0.36 (0-.8_0.07)	0.10
WAZ	Crude model^a^	0.04 (−0.04_0.13)	0.28	−0.01 (− 0.09_0.06)	0.71	− 0.08 (− 0.2_0.04)	0.19	−0.09 (− 0.23_0.04)	0.17
	Adjusted Model^b^	0.002 (0.15_0.15)	0.98	−0.16 (− 0.23_0.3)	**0.02**	− 0.001 (− 0.002_0.00)	**0.03**	−0.18 (− 0.35_-0.004)	**0.04**
HAZ	Crude model^a^	0.009 (−0.08_0.1)	0.85	−0.01 (− 0.11_0.08)	0.76	− 0.08 (− 0.21_0.04)	0.21	−0.04 (− 0.2_-.12)	0.76
	Adjusted Model^b^	0.01 (0.14_0.16)	0.86	−0.003 (− 0.16_0.15)	0.96	0.71 (− 0.002_0.00)	0.2	− 0.14 (− 0.34_0.06)	0.86
BAZ	Crude model^a^	0.07 (−0.04_0.2)	0.21	−0.01 (− 0.12_0.1)	0.85	−0.05 (− 0.22_0.1)	0.49	−0.11 (− 0.3_0.07)	0.85
	Adjusted Model^b^	0.008 (−0.2_0.21)	0.93	−0.24 (0.41_-0.02)	**0.007**	−0.001 (− 0.003_0.00)	0.12	− 0.16 (− 0.4_0.07)	0.08
HCZ	Crude model^a^	0.1 (−0.01_0.05)	0.09	0.04 (−0.08_0.17)	0.47	−0.001 (− 0.002_0.001)	0.24	−0.09 (− 0.06_0.07)	0.22
	Adjusted Model^b^	0.03 (0.19_0.25)	0.78	−0.04 (− 0.06_ − 0.42)	0.07	-0. 01 (− 0.002_0.01)	0.1	−0.001 (− 0.44_0.11)	0.19

*P-value was obtained using linear regression analysis and *P* < 0.05 was considered as significant

*B* Regression coefficient, *CI* Confidence Intervals, *HC* Head Circumference, *WAZ* Weight for Age Z-score, *HAZ* Height for Age Z-score, *BAZ* BMI for Age Z-score, *HCZ* Head Circumference for age Z-score

^a^Row Model: without adjustment of confounding factors

^b^Adjusted model: Adjusted for maternal confounding variables (age, education, occupation, iron and folic acid supplementation status, smoking, maternal illness, pregnancy complications, number of births, history of miscarriage and stillbirth) and infant-related confounding variables (gender, polygamy, gestational week)

The association between maternal pre-pregnancy and pregnancy nutritional adequacy and infants’ anthropometric indices is shown in Table 6. The results showed that there was a significant inverse relationship between maternal nutritional adequacy (both pre-pregnancy and pregnancy status) and weight and WAZ at birth. Head Circumference was inversely associated with pre-pregnancy DDS only in crude model.

## Discussion

To our knowledge, this is the first prospective cohort study to examine the relationship of maternal dietary diversity and nutritional adequacy in both pre- pregnancy and pregnancy condition with anthropometric measurements of infants at birth. In our study, there was no significant association between pre-pregnancy maternal DDS and infant anthropometric indices at birth, but after adjusting for maternal and the neonatal confounders, maternal DDS (in gestational stage) and MAR (in both stages of mother’s intake assessment) were negatively associated with Weight and BAZ.

Total DDS of mothers in pre-pregnancy and pregnancy states were 5.31 and 5.23, respectively. The pre-pregnancy score of the studied women was lower than the amount reported in a cross-sectional study in Tehranian women aged 18–80 years that the mean DDS was 6 [38]. In two study in Bangladesh [39, 40] the mean women diet diversity score (WDDS) of participants who were in the reproductive age was 4.3 and 3.8, respectively that was lower than present study. Another study in Tanzanian pregnant women showed the total score of 3 which was notably lower than other studies [41].

Dietary intake analysis of studied mothers in both state (pre-pregnancy and third trimester of pregnancy) showed that those with higher score for DDS had higher score for intake of all main component of DDS (bread and cereals, meats, dairy, vegetables and fruit groups). In one study that examined WDDS during pregnancy, women with highest total score obtained higher dairy, animal-source foods, and vitamin A rich fruits &vegetables scores than those with less total WDDS [21].

In this prospective cohort study, we observed that maternal DDS during pregnancy was negatively associated with weight, WAZ and BAZ in birth after adjusting for confounding factors. Consistent with this study, the results of a cohort study showed that adequate dietary diversity during pregnancy and greater consumption of dairy, fruits, vegetables, animal foods such as meat and eggs were associated with lower risk of low birth weight (LBW) [21]. Another study showed that Individual Dietary Diversity Score (IDDS) had a significant inverse relationship with LBW incidence (OR = 0.43, *P* = 0.014) [10]. Nonetheless, a randomized controlled trial in India showed that taking of more dairy products, fruits and vegetables before and during pregnancy through a specially formulated snack doesn’t affect birth weight [42]. In another study WDDS of ≥4 food groups during pregnancy was associated with lower risk of maternal anemia, low birth weight (LBW), preterm birth (PTB) [21] . Recently in a study that examined the dietary diversity of pregnant mothers in Tanzania, higher DDS was associated with lower risk of SGA [41]. Maternal nutritional status may be correlated with birth outcomes through affecting the bioavailability of nutrients resources for the fetus and intrauterine growth [43, 44].

In current study, beside maternal and neonatal confounders, adjusting socioeconomic status had an important role in significance of findings. A previous study confirmed that socioeconomic factors, especially household income and education, play a crucial role in women’s dietary diversity and wealthy families and literate women had significantly higher dietary diversity scores [39].

The findings of the present study show that, despite high number of mothers were at the lowest tertile of pre-pregnancy DDS, their frequency at the third tertile was increased in pregnancy stage. Perhaps, one of the possible reasons for increasing the DDS of pregnant mothers was due to receive nutritional counseling from primary health centers/hospitals and increasing their sensitivity to follow the nutritional recommendations during this susceptible period. On the other hand, high percentage of newborns had birth weight in the normal range of 2500 to 4000 g that may be related to increasing mothers’ dietary diversity in the third trimester of pregnancy and improving their weight gain resulted to a normal birth weight range of newborns. However, few studies have examined maternal dietary diversity during pregnancy and its association with neonatal anthropometric indices, and this needs further investigation in future studies.

Generally dietary diversity improves the intake of micronutrients and leads to improved pregnancy outcomes. Non-nutritional factors, such as fetal inflammation due to infection and oxidative stress, epigenetic programming, and maternal stress, can also affect birth outcomes [45, 46]. Particularly in the second trimester of pregnancy, oxidative stress is reached to the highest level and may lead to inflammation and effect on pregnancy outcomes. Obtaining higher dietary diversity score means that variety of antioxidant rich food groups are eaten that may protect against oxidative stress [21]. These factors, unlike maternal dietary diversity, are not easily changed [41].

In the present study, there was no significant relationship between maternal nutritional adequacy before and during pregnancy with neonatal anthropometric measures at birth. After adjusting for maternal and neonatal confounders, a reverse significant relationship was observed between maternal’s nutritional adequacy whit birth weight and WAZ at birth. In a longitudinal cohort study conducted in Australia, women in the highest quartile of MAR had lower fat and saturated fatty acids and higher protein, carbohydrate, and fiber intake compared with women in the lowest quartile. However, there was not any information of their pre-pregnancy intake [47]. In another study examining maternal nutritional adequacy during pregnancy based on RDA, some micronutrients such as vitamin C, vitamin A, potassium, iron and selenium levels were lower than recommended [48].

The present study showed that the average intake of iron, iodine, vitamin B6, vitamin E and vitamin D was lower than recommended dietary allowance (RDA) in pregnant women but the mean calcium intake was 1.4 times higher than RDA. In one study examining the nutrient intake of mothers during pregnancy, the findings showed that mothers had lower vitamin E, folate, magnesium, and iron intake than recommended DRI values [49]. In the Australian study, high percentage of women receiving inadequate calcium, folate, magnesium, and potassium and vitamin E indicating lower nutrient intake than pre-pregnancy RDI. However, these studies did not examine the relationship between maternal nutritional adequacy and neonatal anthropometric indices. In addition, other large studies focusing on maternal nutritional adequacy during pregnancy have also examined only one single nutrient [50, 51]. In a study aimed investigating the effect of pre-pregnancy BMI, energy and nutritional supplements on neonatal body composition, the portion of mother’s carbohydrate from total energy intake was positively related with infant adipose tissue [52].

It seems that high intake of calcium in present study may possibly be associated with reduced risk of overweight and obesity. Apparently, the mechanism of this effect is related to the reduction of PTH and 1, 25-Dihydroxyvitamin D which leads to inhibition of lipogenesis and increase lipolysis and ultimately increase in fecal fat excretion due to the formation of soaps [53]. Perhaps this is a reason for the negative relationship seen in this study. However, this reverse association may be attributed to the fact that mothers with higher adequacy of the diet are at the normal range of weight that affects to gain weight at the normal range during gestation and to deliver a baby with normal birth weight.

The main strength of this study is the information was gathered in a prospective cohort and mothers were followed up during pregnancy until delivery. Also, in order to assess the nutritional status of the mothers, two dietary intake methods were used. 24-h recalls as a gold standard method reflected actual intake of pregnancy stage and FFQ assessed the usual dietary intake of women before deciding to pregnancy. The present study had some limitations include: impossibility of measuring maternal serum micronutrients, measurement errors such as over-reporting and under-reporting of dietary intake [54]. Misreporting may be associated with some factors such as obesity, socioeconomic status, age, depression or poor body image, and health-related behaviors such as smoking or dieting [55, 56]. Inaccurate reporting of exact amounts or types of consumed foods using 24-h dietary recall may be occurred due to dependence of this tool on memory [55]. Also, due to the observational design of the present study, observed inverse association between DDS and anthropometric measurements of newborn at birth may not reflect real causal relationship and should be approved in future experimental/interventional studies.

## Conclusion

Overall, the findings of the study indicate that there is a relationship between maternal dietary diversity during pregnancy and the maternal nutritional adequacy before and during pregnancy with neonatal anthropometric indices at birth. In general, a mother’s nutritional status may have important implications for infant health.

## Data Availability

The datasets analyzed during the current study are not publicly available due to the fact that the used data is part of an ongoing cohort and it is not possible to share the data to the public at this time but are available from the corresponding author on reasonable request.

## Abbreviations

FFQ: Food frequency questionnaire
BMI: Body mass index
WAZ: Weight for age Z-score
HAZ: Height for age Z-score
BAZ: BMI for age Z-score
HCZ: Head circumference for age Z-score
LBW: Low birth weight
MAR: Mean adequacy ratio
NAR: Nutrient adequacy ratio
DDS: Dietary diversity scores
RDA: Recommended dietary allowance
